# Risk factors for infectious complications of ANCA-associated vasculitis: a cohort study

**DOI:** 10.1186/s12882-018-0933-2

**Published:** 2018-06-14

**Authors:** Liu Yang, Honglang Xie, Zhengzhao Liu, Yinghua Chen, Jinquan Wang, Haitao Zhang, Yongchun Ge, Weixin Hu

**Affiliations:** National Clinical Research Center of Kidney Diseases, Jinling Hospital, Nanjing University School of Medicine, Nanjing, 210002 China

**Keywords:** Anti-neutrophil cytoplasmic antibody, Vasculitis, Infection, Lung, Risk factors

## Abstract

**Background:**

Severe infections are common complications of immunosuppressive treatment for antineutrophil cytoplasmic antibody (ANCA)-associated vasculitis (AAV) with renal involvement. We investigated the clinical characteristics and risk factors of severe infection in Chinese patients with AAV after immunosuppressive therapy.

**Methods:**

A total of 248 patients with a new diagnosis of ANCA-associated vasculitis were included in this study. The incidence, time, site, and risk factors of severe infection by the induction therapies were analysed. Multivariate Cox proportional hazards models were used to calculate hazard ratios (HRs) with 95% confidence intervals (CI).

**Results:**

A total of 103 episodes of severe infection were identified in 86 (34.7%, 86/248) patients during a median follow-up of 15 months. The incidence of infection during induction therapy was 38.5% for corticosteroids (CS), 39.0% for CS+ intravenous cyclophosphamide (IV-CYC), 33.8% for CS+ mycophenolate mofetil and 22.5% for CS + tripterygium glycosides, 76 (73.8%) infection episodes occurred within 6 months, while 66 (64.1%) occurred within 3 months. Pneumonia (71.8%, 74/103) was the most frequent type of infection, and the main pathogenic spectrum included bacteria (78.6%), fungi (12.6%), and viruses (8.7%). The risk factors associated with infection were age at the time of diagnosis (HR = 1.003, 95% CI = 1.000–1.006), smoking (HR = 2.338, 95% CI = 1.236–4.424), baseline secrum creatinine (SCr) ≥5.74 mg/dl (HR = 2.153, 95% CI = 1.323–3.502), CD4^+^ T cell< 281 μl (HR = 1.813, 95% CI = 1.133–2.900), and intravenous cyclophosphamide regimen (HR = 1.951, 95% CI =1.520–2.740). Twelve (13.9%) patients died of severe pneumonia.

**Conclusion:**

The infection rate during induction therapy was high in patients with AAV. Bacterial pneumonia was the main type of infection encountered. Age at the time of diagnosis, smoking, baseline SCr ≥5.74 mg/dl, CD4^+^ T cell< 281 μl, and IV-CYC therapy were identified as risk factors for infection.

## Background

Antineutrophil cytoplasmic antibody (ANCA)-associated vasculitis (AAV) is a systemic vasculitis syndrome including microscopic polyangiitis (MPA), granulomatosis with polyangiitis (GPA), eosinophilic granulomatosis with polyangitis (EGPA) and renal-limited vasculitis (RLV). The diagnosis of AAV is based on the presence of clinical manifestations with characteristic histopathological findings and the presence of MPO-ANCA or PR3-ANCA [1–7]. AAV may have predominant involvement of the upper respiratory tract, lungs, kidneys, skin, and nervous system. Most patients with AAV achieved remission after appropriate immunosuppressive therapy with corticosteroids and immunosuppressants, including cyclophosphamide (CYC), mycophenolate mofetil (MMF), and rituximab (RTX) [8–11]. Nevertheless, infection after immunosuppressive therapy contributes to the most common cause of death. The burden of infectious disease in patients with AAV has been reported [1–6, 12–15]. Nonetheless, risk infectors reported so far are inconsistent. In this study, we retrospectively analysed the epidemiological and clinical characteristics of Chinese patients with ANCA-associated vasculitis and discussed major infection episodes occurring during immunosuppressive therapy in a single centre.

## Methods

### Patient selection

A total of 248 patients newly diagnosed with AAV and renal involvement who met the criteria of the Chapel Hill Consensus Conference [7] between January 1, 1998 and December 31, 2013 at the National Clinical Research Center of Kidney Diseases Jinling Hospital were included, among whom 194 patients had renal biopsies that showed pauci-immune necrotic and crescentic glomerulonephritis. All patients were ANCA-positive. Patients with secondary vasculitis, including Henoch-Schonlein purpura, allergy, autoimmune disease, tumour, cryoglobulinemia and infection, were not included. Patients with end-stage renal disease (ESRD) or who received only non-immunosuppressive treatment for infection at the time of diagnosis of AAV were excluded from the study. Ethical statement: This study was approved by the Institutional Review Board of our hospital and performed in accordance with the ethical standards laid down in appropriate version of the Declaration of Helsinki. All patients signed informed consent.

### Clinical and laboratory data

All clinical and laboratory data were collected retrospectively at diagnosis and during the follow-up period, including the patients’ age, gender, medical history, routine blood analysis, 24-h urine protein excretion, urinary sediment red blood cell count, serum albumin and serum creatinine (SCr), liver enzymes, immunoglobulin and T lymphocyte counts, serum ANCAs, lung involvement, Birmingham Vasculitis Activity Score (BVAS) [16], the usage of immunosuppressive agents, methlyprednisone pulse therapy, plasma exchange, and adverse events including major infection. Major infections were diagnosed according to common terminology criteria for adverse events (CTCAE) v4.0 in addition to clinical and radiological manifestations and microorganism cultures.

### Immunosuppressive therapies

None of the patients had received any immunosuppressive therapy before diagnosis. Patients without contraindication initially received intravenous methylprednisolone pulse therapy (0.5 g, once daily, for 3 consecutive days) after diagnosis of AAV. Patients with severe manifestations of AAV underwent plasma exchange therapy. All patients received oral prednisone at a dose of 0.6–0.8 mg/kg/day for 4 weeks, which was then tapered by 5 mg each week to 10 mg/day. Induction immunosuppressive agents included MMF 1–1.5 g/day orally, monthly intravenous cyclophosphamide (IV-CYC) at 0.75–1.0 g/m^2^ body surface area in monthly pulses, tripterygium glycosides (TW, extract from the traditional Chinese herb *Tripterygium wilfordii,* which mainly contains triptolide) and multi-target therapy (prednisone, mycophenolate mofetil and tacrolimus) [8]. Maintenance therapy included prednisone 5 mg/day combined with MMF and azathioprine. Prophylaxis of *Pneumocystis Jirovecii* pneumonia (PJP) with SMZ-CO (trimethoprim-sulfamethoxazole 400/80 0.48 g per day) was used in patients whose CD4^+^ T cell counts were less than 200/μl, and the doses were tapered in patients with renal dysfunction [17].

### Antimicrobial therapy

All immunosuppressive agents, except prednisone, were discontinued in patients with AAV who suffered from major infection during the follow-up period. Antimicrobial therapy was prescribed according to clinical and radiological manifestations and microbiological characteristics. Patients diagnosed with PJP were treated with SMZ-CO and echinocandin together.

### Supportive therapy

Patients with weight loss were prescribed enteral nutrition. The patients with severe acute kidney injury or acute respiratory distress syndrome (ARDS) were treated with continuous blood purification.

### Definitions

A recorded severe infectious complication was defined as implying the administration of an antimicrobial medication for an observable clinical, microbiological and radiologic suspected infection requiring hospitalization. Immediate dialysis was defined as the clinical necessity of renal replacement therapy on admission. The first immunosuppressive agent used in addition to corticosteroids was termed induction therapy. The immunosuppressive regimen used during follow-up was termed the maintenance agent. The diagnosis criteria for deep fungal infection included clinical manifestations, such as fever, cough, diarrhoea or lower urinary tract symptoms, and the detection of fungi in sputum, urine, stool or tissue specimens. Cytomegalovirus (CMV) infection was diagnosed by CMV polymerase chain reaction (PCR). The range of quantification of this assay was 600–100,000 copies/ml for CMV. CMV pneumonia was defined as the detection of ground glass opacity by chest X-ray film or computed tomography, the detection of CMV in the bronchoalveolar lavage fluid or lung tissue samples, and clinical signs such as fever, cough, dyspnoea and hypoxemia. The diagnosis of PJP was made clinically or by the identification of *Pneumocystis* from sputum, bronchoalveolar fluid, tracheal secretions or lung tissue by special stains or a non-nested PCR, specifically designed to diagnose pneumonia rather than colonization [18]. ARDS was defined as the acute onset of hypoxemia (arterial partial pressure of oxygen to fraction of inspired oxygen [P_aO2_/F_IO2_] ≤ 200 mmHg) with bilateral infiltrates on chest radiographs, without left atrial hypertension. Multiple organ dysfunction syndrome (MODS) was defined as the simultaneous failure of at least two organs. ESRD was defined as eGFR < 15 ml/min per 1.73m^2^ or requiring renal replacement treatment for > 3 months.

### Follow-up and endpoints

The follow-up endpoints included the final date of December 31, 2014, dropping out before the final date, reaching ESRD, or death.

### Statistical analysis

Statistical analysis was performed with SPSS 20.0 for Windows (SPSS Inc., Chicago, IL, USA). Medians and ranges were reported for non-normally distributed data, and means ± standard deviations were reported for normal-distributed data. The Kruskal-Wallis test was applied for the comparison of non-normal distributed data. Differences between means were tested using the Student’s t-test. A Mann-Whitney U test was used for nonparametric distributions. Chi-squared tests were used for the comparison of categorical data. To address the independent predictive value of factors associated with the rate of infections, the variables with *P* values of less than 0.1 in univariate analysis as well as those reported in the literature were selected for multivariate analysis using the Cox regression model. The group with corticosteroids only was used as a reference group in multivariate analysis. Only the time to first severe infection was evaluated. Laboratory values and BVAS used for modelling were from the time of diagnosis. Receiver operating characteristic (ROC) curve analysis was performed to determine the cut-offs of SCr, haemoglobin, albumin, CD4+ T cells and BVAS. All tests were two-tailed, and *P*-values of < 0.05 were considered significant. Confidence intervals (CIs) were calculated at the 95% level.

## Results

### Characteristics of the cohort

This study identified 248 individuals with ages ranging from 14 to 78 years (median 55 years), including 214 cases diagnosed as MPA, 16 cases diagnosed as RLV, 10 cases diagnosed as GPA and 8 cases diagnosed as EGPA. Seventy-five patients (30.2%) showed lung involvement, 30 (12.1%) had alveolar haemorrhage, and 54 (21.8%) had sinus involvement. MPO-ANCA was more prevalent, and only 21 cases (8.5%) were PR3-ANCA-positive. Fifty-three patients started immediate dialysis. Initial immunosuppressive treatment consisted of pulse methylprednisolone (67.3%), plasma exchange (23.8%), IV-CYC (26.6%), MMF (31.0%) and TW (16.1%). Twenty-six percent of patients received only oral corticosteroids (Table 1). Forty-two patients (16.5%) received SMZ-CO to prevent PJP, and 29 of them were CYC users.

**Table 1:** Clinical characteristics of AAV patients complicated with or without infection

	AAV (*n* = 248)	Infection group (*n* = 86)	Non-infection group (*n* = 162)	*P*
Age(ys)	55.0 (42.8~ 64.0)	58.0 (46.8~ 66.0)	54.0 (40.0~ 63.0)	0.082
male, n(%)	103 (41.5)	40 (46.5)	63 (38.9)	0.155
smoke, n(%)	59 (23.8)	31 (36.0)	28 (17.3)	0.000**
Diabetes, n(%)	11 (4.4)	7 (8.1)	4 (2.5)	0.032*
MPO-ANCA, n(%)	227 (91.5)	79 (91.9)	148 (91.4)	0.968
PR3-ANCA, n(%)	21 (8.5)	7(8.1)	14 (8.6)	0.968
Hemoglobin (g/dl)	8.5 (7.4~ 9.7)	8.2 (6.7~ 9.2)	8.8 (7.7~ 9.9)	0.004**
White blood cell (/mm3)	7200.0 (5000.0~ 10,000.0)	7600.0 (5900.0~ 11,350.0)	6900.0 (4900.0~ 9750.0)	0.049*
Albumin (g/l)	35.5 (31.6~ 38.7)	33.8 (30.3~ 36.9)	35.9 (32.4~ 39.6)	0.002*
Globulin (g/l)	27.4 (23.4~ 32.2)	26.8 (22.3~ 33.1)	27.6 (23.5~ 31.6)	0.761
Creatinine (mg/dl)	3.3 (1.8~ 5.3)	4.16 (2.5~ 6.9)	2.8 (1.5~ 4.7)	0.000**
eGFR< 60 ml/min per 1.73m^2^, n(%)	216 (87.1)	81 (94.2)	135 (83.3)	0.000**
CD4 lympnocyte cell(/ul)	416.0 (234.5~ 589.8)	399.0 (207.5~ 552.0)	428.0 (258.0~ 662.0)	0.027*
IgG (g/l)	13.3 (10.4~ 16.4)	13.4 (10.4~ 16.5)	12.9 (10.3~ 16.3)	0.570
C3 (g/l)	0.9 (0.8~ 1.1)	0.9 (0.8~ 1.2)	0.9 (0.8~ 1.1)	0.569
Lung involvement, n(%)	75 (30.2)	34 (39.5)	41 (25.3)	0.011**
BVAS	14 (12~ 16)	15 (12~ 17)	14 (12~ 16)	0.009**
MP pulse therapy, n(%)	167 (67.3)	56 (65.1)	111 (68.5)	0.929
Plasma exchanage, n(%)	59 (23.8)	25 (29.1)	34 (20.9)	0.109
Induction therapy				
corticosteroids only, n(%)	65 (26.2)	25 (29.1)	40 (24.7)	0.349
corticosteroids+CYC, n(%)	66 (26.6)	26 (30.2)	44 (27.2)	0.938
corticosteroids+MMF, n(%)	77 (31.0)	26 (30.2)	47 (29.0)	0.245
corticosteroids+TW, n(%)	40 (16.1)	9 (10.5)	31 (19.1)	0.052

**P < 0.01; *P < 0.05, *IgG* Immunoglobulin G, *BVAS* Birmingham vasculitis activity score, *MP* Methlyprednisone, *CYC* Cyclophosphamide, *MMF* Mycophenolate mofetil

### Incidence and location of infection

A total of 103 infectious episodes occurred in 86 patients (34.7%) during follow-up for 1~ 155 months (median 15 months). Fifteen cases experienced a second episode of infection, and one patient experienced a third episode. Seventy-six episodes (73.8%) of infection occurred during induction therapy (median 1.5 months). Twenty-seven episodes (26.2%) occurred during maintenance therapy (median 18 months), and six episodes (5.8%) occurred after 24 months. Pulmonary infections (71.8%, 74/103) were the most frequent type of infection, followed by skin (*n* = 7, 6.8%), digestive tract (*n* = 3, 2.9%), urinary tract (*n* = 2, 1.9%) and central nervous system (*n* = 1, 1.0%) infections. Six patients (5.8%) developed sepsis because of their reported infection.

### Pathogens

The pathogens responsible for infection were confirmed in 87 episodes. The whole pathogen spectrum included bacteria, fungi and viruses. Bacterial infection was the most common (*n* = 57, 66%), especially *Acinetobacter baumannii*, followed by fungal (*n* = 21, 24%) and viral infections (*n* = 9, 10%) There were four CMV, seven PJP and 16 unspecified infections (Table 2).

**Table 2:** Pathogens of infection

	Pathogens (*n* = 87)	N (%)
Bacterium	Acinetobacter baumannii	6 (7)
	*Staphylococcus aureus*	5 (6)
	Pseudomonas aeruginosa	3 (4)
	*Escherichia coli*	2 (2)
	*Klebsiella pneumoniae*	2 (2)
	Neisseria	2 (2)
	M.tuberculosis	2 (2)
	Viridans Streptococci	1 (1)
	Streptococcus	1 (1)
	Salmonella enteritidis	1 (1)
	CitrobacterWerkmanandGillen	1 (1)
	A.juniiBouvetandGrimont	1 (1)
	Stenotrophomonas maltophilia	1 (1)
	*Enterobacter cloacae*	1 (1)
	Nonspecific infection	28 (32)
Fungus	C.albicans	9 (11)
	Pneumocystis jiroveci	7 (8)
	Aspergillus fumigatus	3 (4)
	*Candida tropicalis*	2 (2)
Virus	Varicella-zoster virus	5 (6)
	Cytomegalovirus	4 (5)

### Risk factors for infection

The infectious rate of induction therapy with corticosteroids only was 38.5% (25/65), that for CS + IV-CYC was 39.0% (26/66), CS + MMF was 33.8% (26/77) and CS + TW was 22.5% (9/40). The incidence of smoking (36.0% vs. 17.3%, *P* = 0.000) and diabetes (8.1% vs. 2.5%, *P* = 0.032) was significantly higher among the infected patients. The cutoff level of SCr haemoglobin, albumin, CD4+ T cells, and BVAS were determined as 5.74 mg/dl, 7.75 g/dl, 33.95 g/l, 281/ul, and 25.5 respectively based on ROC curve analysis. Single factor analysis revealed that risk factors for complicated infection in patients with AAV included age, smoking, pulmonary involvement, hemoglobulin, albumin, SCr level, CD4 ^+^ T cell count, BVAS, and immunosuppressive therapy with MMF, CYC and TW. In adjusted models for the AAV cohort, increased risks of infection were observed in patients who were older at the time of diagnosis (HR = 1.003, 95% CI = 1.000–1.006), smoking (HR = 2.338, 95% CI = 1.236–4.424), with baseline Scr ≥5.74 mg/dl (HR = 2.153, 95% CI = 1.323–3.502), CD4^+^ T cell< 281 μl (HR = 1.813, 95% CI = 1.133–2.900), and users of intravenous cyclophosphamide regimen (HR = 1.951, 95% CI =1.520–2.740) (Table 3).

**Table 3:** COX regression for AAV complicated infection

	Single factor analysis		Multiple factor analysis	
varium	HR (95% CI)	P	HR (95% CI)	P
Age	1.004 (1.001–1.007)	0.008	1.003 (1.000–1.006)	0.030
gender	1.444 (0.938–2.221)	0.095	0.723 (0.394–1.328)	0.296
smoking	2.293 (1.465–3.588)	0.000	2.338 (1.236–4.424)	0.009
Diabetes	1.504 (0.651–3.474)	0.339		
Hemoglobin< 7.75 g/dl	2.079 (1.358–3.182)	0.001	1.362 (0.827–2.243)	0.224
Albumin< 33.95 g/l	1.902 (1.243–2.910)	0.003	1.178 (0.740–1.874)	0.490
baseline creatinine higher than 5.74 mg/dl	3.190 (2.053–4.957)	0.000	2.153 (1.323–3.502)	0.002
CD4^+^T cell< 281/ul	02.021 (1.316–3.105)	0.001	1.813 (1.133–2.900)	0.013
BVAS at the time of diagnosis > 25.5	1.883 (0.815–4.349)	0.138		
corticosteroids+MMF	1.945 (1.156–3.272)	0.012	1.004 (0.571–1.765)	0.989
corticosteroids+CYC	1.906 (1.073–3.383)	0.028	1.951 (1.520–2.740)	0.042
corticosteroids+TW	1.519 (1.110–2.715)	0.042	0.572 (0.262–1.250)	0.161

*MP* Methlyprednisone, *MMF* mycophenolate mofetil, *CYC* cyclophosphamide, *TW* Tripterygium wilfordii

### Characteristics of pneumonia

The exact pathogen was identified in 44 of 82 episodes of pneumonia. Bacteria were the most common pathogens (*n* = 27, 61.4%), especially *Acinetobacter baumannii* (*n* = 6, 13.6%), *Staphylococcus aureus* (*n* = 5, 18.5%) and *Pseudomonas aeruginosa* (*n* = 3, 6.0%). Thirteen cases were diagnosed as fungal infections, and most were caused by *C. albicans* (*n* = 8, 61.5%). CMV was identified in all four cases with viral pneumonia.

The main pulmonary radiologic findings included consolidation (*n* = 38, 51.4%), diffuse interstitial pneumonia (*n* = 21, 28.4%) and multiple nodules (*n* = 13, 17.6%). Bacterial pneumonia presented with consolidation (*n* = 24, 32.4%), nodules (*n* = 9, 12.2%) and a diffuse reticular pattern (*n* = 6, 8.1%). CMV pneumonia mainly presented with ground-glass opacities (4, 5.4%), diffuse reticular thickening (*n* = 3, 4.1%) and nodules (*n* = 1, 1.4%) on bilateral lungs. Fungal pneumonia was characterized by consolidation (*n* = 14, 18.9%), nodules (*n* = 4, 5.4%), halo (*n* = 4, 5.4%) and air crescent sign (*n* = 2, 2.7%). Thirteen cases were complicated by ARDS, and 10 were complicated by MODS. Nine patients required mechanical respiration (5 BiPAP and 4 endotracheal intubation).

### Treatment and outcome of infectious episodes

All 103 episodes were treated with intravenous antibiotics. Twelve (11.7%) of 103 patients died and all due to severe pneumonia. The time to death was from one to sixteen months after the initiation of immunosuppressive therapy. None died due to AAV (Table 4).

**Table 4:** ESRD and Death

	Infection group	Non-infection group
ESRD	21	13
Time to ESRD, (months)	2.5 (1~ 7)	12 (5~ 32.5)
Death	12	0
Time to Death, (months)	3 (2~ 12)	
Cause of Death		
Acinetobacter baumannii	1	
Staphylococcus aureus	1	
Stenotrophomonas maltophilia	1	
C.albicans	1	
Pneumocystis jiroveci	1	
Aspergillus fumigates	1	
Nonspecific infection	1	

*ESRD* end-stage renal disease

## Discussion

A link between vasculitis and infection has long been suspected. Bacterial infections can trigger the production of various autoantibodies, including ANCA [19]. Infection is a major concern in the management of AAV and is the most common cause of death, especially in patients with malnutrition or immunosuppressive therapy [1–3]. Immunosuppressive therapy is performed with consideration of the disease activity, which is comprehensively evaluated based on the BVAS score [20]. Nonetheless, even in patients with severe ANCA-associated vasculitis, secondary infection, rather than active AAV, is the leading cause of death [21]. There still remains no firm conclusion about the burden and characteristics of major infections in patients with AAV.

We retrospectively reviewed the clinical charts of 248 Chinese patients with AAV. Major infections were reported in 34.6% of our single-centre cohort. Approximately 64.1% of these infections developed in the first three months of induction therapy. In the reported studies, corticosteroids contributed to 89% of infections of patients with AAV, and the infection rate decreased when the corticosteroids were tapered [1–4]. Corticosteroid treatment leads to an immunocompromised status in patients by inhibiting cytokines, neutrophils, and immunologic response and by exerting anti-inflammatory and immunosuppressive effects [22]. Infection is suspected when fever (≥37.3 °C) persists for no less than three days and C-reactive protein increases after remission of AAV [22]. The evaluation of infection is based on the presence of organ manifestations. Identificating methods of causative microorganisms, such as common bacteria, viruses, and fungi, include mycological, histological, and genetic tests [20].

The main areas of infection included the lungs and skin. The lung infection rate was as high as 79.6% in this cohort. Most AAV patients had impaired renal function, and lung involvement and diffuse alveolar haemorrhage injure the local protective barrier. Renal injury also increases the risk of severe infection and is closely associated with a poor outcome [6, 18]. According to the literature, the most common causative pathogens are bacteria, such as *Streptococcus pneumonia* and *Haemophilus influenza* [23, 24], followed by fungi and viruses. In our cohort, the main bacteria included *Acinetobacter baumannii*, *Staphylococcus aureus*, and *Pseudomonas aeruginosa* (Table 2), and our rates of infections with fungi and viruses were higher and lower, respectively, than those of previous reports [25]. Characteristics of AAV, such as global inflammation, renal injury, lung involvement, malnutrition, and immunosuppressive therapy, contribute to infections by opportunistic pathogens [26]. CMV, PJP and 13 cases of pneumomycosis developed during induction therapy. It is also possible that a PJP diagnosis may have been missed, especially earlier in the study period when our microbiology laboratory used Pneumocystis stains. Some clinical studies have concluded that *Streptococcus pneumonia* and influenza vaccines are safe and effective [27–29]. Thus, improving the vaccination coverage against streptococcus pneumonia and influenza in high-risk populations could play an important role in pulmonary prevention [30].

The most common computed tomography findings were ground-glass attenuation, reticular pattern, and fibrous bands with infiltration. In cases of bacterial, fungal and viral pneumonia, a consolidation and reticular pattern, patchy consolidation and glass-ground attenuation were most commonly observed, respectively. These characteristics are predominantly seen in pneumonia patients with AAV.

Given the high incidence of infections in patients with AAV, risk factors need to be defined in order to increase surveillance and prescribe prophylactic antibiotic therapy. Many studies have reported that age, female gender, diabetes, impaired renal function, clinical grade category of rapidly progressive glomerulonephritis (RPGN), lymphopenia and immunosuppressive therapy are risk factors for infection in AAV [6, 12–14, 20, 31]. However, there remains no consensus about the infectious risk factors in Chinese patients with AAV. In this cohort, BVAS and the frequency of diabetes in the infectious group were higher than that in the control group, indicating that higher BVAS and diabetes are potential risk factors of infection. Age at the time of diagnosis (HR = 1.003, 95% CI = 1.000–1.006), smoking (HR = 2.338, 95% CI = 1.236–4.424), baseline Scr ≥5.74 mg/dl (HR = 2.153, 95% CI = 1.323–3.502), CD4^+^ T cell< 281 ul (HR = 1.813, 95% CI = 1.133–2.900), and use of intravenous CYC were independent risk factors of infection. Whether or not the use of CYC was a risk factor for developing infection in AAV patients remains controversial [9, 10, 14]. Masaharu [20] also reported that the use of CYC was a risk factor for developing infection in AAV patients, but no difference was observed in renal failure between those with or without infection. On the other hand, CYC showed similar adverse events when compared to Rituximab in two randomized controlled trials [9, 10].

In our study, the infection-related mortality (11.7%) was less than that reported in most of the literatures [4, 13, 14, 32]. Half of these cases died within the first month after diagnosis. Thus, clinicians should consider adaptive immunosuppressive agents to avoid life-threatening infection.

### Limitations

There are some limitations in this retrospective study. First, the treatment protocols were not uniform and lack of data on Rituximab. Only a minority of patients were given SMZ-CO prophylaxis because of the insufficient awareness. None of these patients received prophylaxis for fungal infection. In addition, some cases with pulmonary or central nervous system infection failed to show a definitive pathogen. The frequency and severity of pneumonia should be lowered by prophylactic treatment and early diagnosis.

## Conclusion

Infections can develop during every stage of AAV, primarily in the lungs and skin. The pathogens identified in this study mainly consisted of bacteria, candidiasis, CMV and herpes simplex virus, and age at the diagnosis, smoking, baseline SCr higher than 5.74 mg/dl, CD4^+^ T cell< 281 μl, and CYC therapy were independent risk factors for infection in patients with AAV.

## Abbreviations

AAV: Antineutrophil cytoplasmic antibody -associated vasculitis
ANCA: Antineutrophil cytoplasmic antibody
ARDS: Acute respiratory distress syndrome
BVAS: Birmingham Vasculitis Activity Score
CI: Confidence intervals
CMV: Cytomegalovirus
CS: Corticosteroids
CTCAE: Common terminology criteria for adverse events
CYC: Cyclophosphamide
EGPA: Eosinophilic granulomatosis with polyangitis
ESRD: End-stage renal disease
GPA: Granulomatosis with polyangiitis
HRs: Hazard ratios
IV-CYC: Intravenous cyclophosphamide
MMF: Mycophenolate mofetil
MODS: Multiple organ dysfunction syndrome
MPA: Microscopic polyangiitis
PCR: Polymerase chain reaction
PJP: *Pneumocystis Jirovecii* pneumonia
RLV: Renal-limited vasculitis
ROC: Receiver operating characteristic
RTX: Rituximab
SCr: Secrum creatinine
SMZ-CO: trimethoprim-sulfamethoxazole 400/80
TW: Tripterygium glycosides
